# The Number of Chronic Conditions and Domain-Specific Movement Behaviours Among People Aged ≥15 Years: Findings from the 2019 Serbian National Health Survey

**DOI:** 10.3390/healthcare14172783

**Published:** 2026-09-01

**Authors:** Dragana Jovic, Jadranka Plavsic, Shihab Aldin Ahmad Al-Riyami, Maja Vukovic, Veroslava Stankovic, Jasmina Pluncevic Gligoroska

**Affiliations:** 1 Institute of Public Health of Serbia “Dr. Milan Jovanović Batut”, Dr. Subotića Starijeg 5, 11000 Belgrade, Serbia; 2 Serbian Institute of Sport and Sports Medicine, Kneza Višeslava 72, 11000 Belgrade, Serbia; jadranka.plavsic@rzsport.gov.rs; 3 Department of Sport Activities, Sultanate of Oman Sultan Qaboos University, Muscat 123, Oman; shihab_volly@hotmail.com; 4 Faculty of Medicine Foča, University of East Sarajevo, Studentska 5, 73300 Foca, Bosnia and Herzegovina; majavukovic81@gmail.com; 5 The College of Health Science, Academy of Applied Studies, Cara Dušana 254, 11000 Belgrade, Serbia; stankovicveroslava@gmail.com; 6 Faculty of Medicine, University Ss. Cyril and Methodius, 50th Division Street 6, 1000 Skopje, North Macedonia; jasnapg965@yahoo.com

**Keywords:** chronic disease, multimorbidity, domain-specific physical activity, sedentary behaviour, functional mobility

## Abstract

Background/Objectives: Previous studies have linked chronic conditions and multimorbidity to lower physical activity, but evidence across individual movement behaviours remains limited. We examined associations between the number of self-reported chronic diseases and conditions and transport-related walking, leisure time physical activity, muscle-strengthening activity and sedentary behaviour among people aged ≥15 years in Serbia. Methods: Nationally representative 2019 Serbian National Health Survey data were analysed (*n* = 13,076 with complete information on 17 conditions). Complex sample logistic regression models were sequentially adjusted for sociodemographic characteristics, body mass index, functional mobility and self-rated health. Additional analyses assessed linear trends and departures from linearity using all covariates except self-rated health. Results: Overall, 51.4% reported no condition, 18.0% one and 30.6% two or more. All outcomes were associated with condition status before adjustment, but most associations were attenuated after including functional mobility. After full adjustment, an overall association remained only for muscle-strengthening activity on <2 days/week (Wald F = 5.13; *p* = 0.006). Compared with no condition, one condition was associated with higher odds (aOR 1.60, 95% CI 1.18–2.16), but two or more were not. Each additional condition was associated with 4.5% higher odds of sitting or reclining for ≥6 h/day (aOR 1.045, 95% CI 1.006–1.087; *p* = 0.025). No other linear trends were identified; muscle-strengthening activity showed a departure from linearity (*p* < 0.001). Conclusions: Associations differed across movement behaviour domains and were attenuated particularly after including functional mobility. The number of conditions alone only partially accounted for observed differences in movement behaviours.

## 1. Introduction

Chronic non-communicable diseases constitute a major public health concern and are associated with substantial morbidity, disability and premature mortality worldwide [1]. Population ageing, longer life expectancy and the rising prevalence of chronic diseases have been accompanied by a greater frequency of multiple long-term conditions occurring within the same individual [2,3,4]. Although multimorbidity is more common in later life, it is also reported among younger and middle-aged adults and may have lasting implications for health and functioning across the adult life course [2,3,4]. Its presence has been associated with poorer quality of life, functional limitations, more complex healthcare needs and greater use of health services [2,3,4].

Regular physical activity is widely recommended for the prevention and management of chronic diseases and for the preservation of physical functioning and quality of life [5,6,7]. Although insufficient physical activity is recognised as contributing to the development of several chronic diseases, opportunities for physical activity may, in turn, be restricted by the presence of multiple conditions through symptoms, impaired physical capacity and functional limitations [7,8]. The relationship between chronic disease and movement behaviour may therefore be complex and potentially bidirectional, particularly when several conditions coexist.

Population-based evidence suggests that the association between multiple chronic conditions and physical activity is not restricted to older adults, although its strength and consistency may vary across the adult life course. In a Spanish study of 22,190 adults aged ≥16 years, an inverse association between multimorbidity and physical activity was identified in the youngest and oldest age groups, while functional limitations were associated with lower activity levels predominantly among adults aged ≥45 years [9]. In a multinational study of 228,024 adults aged ≥18 years from 46 low- and middle-income countries, multimorbidity was associated with insufficient physical activity in the overall population, although stronger and more consistent associations were observed among those aged ≥50 years [10]. Similarly, in a nationally representative sample of 36,697 United States adults aged ≥18 years, progressively lower odds of achieving the recommended level of aerobic leisure time physical activity were observed with each additional chronic condition [11].

Current World Health Organization guidelines recommend that adults, including those living with chronic conditions, undertake 150–300 min of moderate-intensity or 75–150 min of vigorous-intensity aerobic physical activity each week, together with muscle-strengthening activities involving all major muscle groups on at least two days per week [5]. Muscle-strengthening activity has also been associated with health benefits independently of aerobic physical activity, supporting its consideration as a distinct component of health-enhancing movement [12].

In population health research and surveillance, physical activity is frequently summarised using a measure of total volume or adherence to overall recommendations, although it is undertaken across distinct domains and in different contexts [13,14]. Transport-related walking may form part of everyday mobility, whereas leisure time and muscle-strengthening activities may be more discretionary and dependent on available time, motivation, physical capacity and access to supportive environments. Sedentary behaviour represents a separate component of daily movement and cannot be inferred solely from participation in physical activity [5,15,16,17]. Because movement behaviours differ in the contexts in which they occur and in their physical demands, their associations with the number of chronic conditions may not be uniform.

However, population-based evidence on these domain-specific associations remains limited, particularly in Central and Eastern Europe. Transport-related walking, leisure time physical activity, muscle-strengthening activity and sedentary behaviour have rarely been examined concurrently within the same nationally representative population while accounting for sociodemographic characteristics, functional mobility and self-rated health. Therefore, the associations between the number of self-reported chronic diseases and conditions and these four movement behaviours were examined among people aged ≥15 years in Serbia. Linear trends and possible departures from linearity were also assessed.

## 2. Materials and Methods

### 2.1. Study Design and Data Source

Data from the 2019 Serbian National Health Survey, conducted as part of the third wave of the European Health Interview Survey (EHIS wave 3), were used in this cross-sectional study [18,19]. The survey was jointly implemented by the Statistical Office of the Republic of Serbia, the Institute of Public Health of Serbia “Dr Milan Jovanović Batut” and the Ministry of Health of the Republic of Serbia, in accordance with standardised EHIS methodology [18,19]. Nationally representative health information was collected for the population of the Republic of Serbia and its four statistical regions: Belgrade, Vojvodina, Šumadija and Western Serbia, and Southern and Eastern Serbia [18].

In accordance with the EHIS definition, the target population comprised individuals aged ≥15 years who were usual residents of private households in the Republic of Serbia [18,19]. Individuals living in collective households or institutional settings—including student residences, residential care facilities, homes for older people or adults with disabilities, monasteries and similar institutions—were not included in the sampling frame [19]. Data were collected between October and December 2019 [18].

A stratified two-stage cluster sampling design was applied [18]. Stratification was based on the four statistical regions and type of settlement, classified as urban or other. The 2011 Census of Population, Households and Dwellings served as the sampling frame. In the first stage, census enumeration areas were selected as primary sampling units by systematic probability-proportional-to-size sampling, with the number of households in each enumeration area used as the measure of size. Within each stratum, enumeration areas were ordered by municipality and identification number, thereby providing implicit geographical stratification. In the second stage, households were selected with equal probability by simple random sampling from census household lists [18].

Data were collected using standardised questionnaires and objective measurements [18,19]. Face-to-face interviews were conducted using computer-assisted personal interviewing, while a separate self-administered questionnaire was used for sensitive topics. Height, body weight and blood pressure were measured among household members aged ≥15 years. Fieldwork was undertaken by trained teams comprising one interviewer and one healthcare professional. The variables examined in the present study were obtained from the face-to-face individual questionnaire, except for body mass index, which was calculated from measured height and body weight [18].

Of 13,589 eligible household members aged ≥15 years, 13,178 completed the individual interview, corresponding to an individual response rate of 97.0%; the household response rate was 80.7% [18]. Complete information on all 17 chronic diseases and conditions examined in the present study was available for 13,076 participants (99.2% of the interviewed sample), while information on functional mobility status was available for 13,161 participants. Because data availability differed across movement behaviour outcomes and covariates, analyses were restricted to participants with complete information on the variables included. Consequently, the unweighted analytical sample size differed between outcomes but remained constant across regression models within each outcome.

### 2.2. Assessment of Chronic Disease and Condition Status

Chronic disease and condition status was assessed using the disease-specific morbidity questions included in the 2019 Serbian National Health Survey [18,19]. Participants were asked whether they had experienced each listed disease or condition during the preceding 12 months. Sixteen items corresponded to the standard EHIS wave 3 disease-specific morbidity module, while malignant disease was included as an additional item in the Serbian survey [18,19]. The 17 assessed diseases and conditions comprised asthma, including allergic asthma; chronic bronchitis, chronic obstructive pulmonary disease or emphysema; myocardial infarction or its chronic consequences; coronary heart disease or angina pectoris; hypertension; stroke or its chronic consequences; arthrosis or degenerative joint disease; chronic low-back disorder; chronic neck disorder; diabetes mellitus; allergy, excluding allergic asthma; liver cirrhosis; urinary incontinence or bladder control problems; kidney problems; depression; high blood cholesterol or high blood lipids; and malignant disease.

Responses to each item were classified as indicating the presence or absence of the respective disease or condition. Responses recorded as ‘do not know’, refusal or unavailable were treated as missing. Chronic disease and condition status was determined only for participants who provided valid responses to all 17 items. The number of self-reported diseases and conditions was calculated for each participant by summing the conditions reported as present, yielding a possible range from 0 to 17.

For descriptive analyses, participants were classified as reporting no disease or condition, one, two, three, or four or more diseases and conditions. For the primary regression analyses, chronic disease and condition status was categorised as none, one, or two or more diseases and conditions. In additional analyses, the number of self-reported chronic diseases and conditions was entered as a continuous variable to assess linear trends and possible departure from linearity.

### 2.3. Assessment of Domain-Specific Movement Behaviours

Four domain-specific movement behaviours were assessed: transport-related walking, leisure time physical activity, muscle-strengthening activity and sedentary behaviour. Transport-related walking, leisure time physical activity and muscle-strengthening activity were assessed using the relevant items from the European Health Interview Survey Physical Activity Questionnaire (EHIS-PAQ), as administered in the 2019 Serbian National Health Survey [18,19,20,21].

Transport-related walking was assessed from the reported number of days in a typical week on which participants walked for transport for at least 10 consecutive minutes and the time usually spent walking for transport on such a day [19]. For the purposes of the present study, regular transport-related walking was defined as walking for transport on at least five days per week for at least 30 min on a typical day. Participants who did not meet both criteria were classified as not reporting regular transport-related walking.

Leisure time physical activity comprised sports, fitness and recreational activities undertaken for at least 10 consecutive minutes and resulting in at least a small increase in breathing or heart rate [19]. Participants reporting no such activity during a typical week were classified as leisure time physically inactive, while those reporting any participation were classified as engaging in leisure time physical activity. This classification was used to distinguish non-participation from any participation. The resulting measure reflected participation status and was not intended to represent adherence to physical activity recommendations.

Muscle-strengthening activity was assessed from the reported number of days in a typical week on which activities specifically intended to strengthen the muscles, such as resistance training or strength exercises, were undertaken [19]. Participants were classified as undertaking muscle-strengthening activity on fewer than two days or on at least two days per week. The threshold of at least two days per week was selected in accordance with current recommendations for muscle-strengthening activity [5].

Sedentary behaviour was assessed using the EHIS question on the total time spent sitting or reclining during a typical day, including time spent at work, at home, travelling or socialising, but excluding sleep [19]. The original response categories were combined to classify participants as spending either less than six hours or at least six hours per day sitting or reclining. This categorisation was used for analytical purposes and was not intended to denote an established clinical threshold.

### 2.4. Covariates

Covariates were selected a priori on the basis of their potential relationships with chronic disease burden and movement behaviours, their relevance to the study question, and their availability and completeness in the survey database. Covariates included sex, age, educational attainment, body mass index (BMI), region of residence, household income quintile, functional mobility status and self-rated health. Age was categorised as 15–29, 30–44, 45–64 or ≥65 years.

Educational attainment was classified as primary education or lower, secondary education, or higher education, in accordance with the International Standard Classification of Education 2011 [22]. BMI was calculated from measured height and body weight and categorised as <25.0, 25.0–29.9 or ≥30.0 kg/m^2^, using the World Health Organization BMI thresholds [23].

Region of residence and household income quintile were obtained as predefined variables from the 2019 Serbian National Health Survey database [18]. Region of residence was classified as Belgrade, Vojvodina, Šumadija and Western Serbia, or Southern and Eastern Serbia. Household income quintile ranged from the first (lowest income) to the fifth (highest income) quintile.

Functional mobility was assessed from reported difficulty walking 500 m on level ground without assistance and walking up or down 12 steps [18,19]. Participants were classified as having no difficulty with either task, difficulty with one task, or difficulty with both tasks. Self-rated health was assessed using the standard five-category EHIS item and was categorised as very good/good or fair/bad/very bad [18,19].

### 2.5. Ethical Considerations

The 2019 Serbian National Health Survey was conducted in accordance with the principles of the Declaration of Helsinki and national ethical and data protection requirements [18,24]. Ethical approval was granted by the competent territorial ethics committees representing the four main regions of Serbia, under the coordination of the Institute of Public Health of Serbia “Dr Milan Jovanović Batut” [18]. Written informed consent was obtained from all participants before their inclusion in the survey. Anonymised survey data were used for the present secondary analysis.

### 2.6. Statistical Analysis

Statistical analyses were performed using IBM SPSS Statistics for Windows, version 26.0 (IBM Corp., Armonk, NY, USA). The complex multistage sampling design of the 2019 Serbian National Health Survey was accounted for in all analyses using the Complex Samples module. Sampling weights, strata and primary sampling units were incorporated to obtain estimates representative of the population of Serbia aged ≥15 years and to produce standard errors that reflected the sampling design.

Participant characteristics, the prevalence of individual diseases and conditions, and movement behaviours were summarised using survey-weighted percentages with 95% confidence intervals (CIs). Sample sizes were reported as unweighted counts. Differences in categorical distributions were assessed using second-order Rao–Scott-adjusted Pearson chi-squared tests.

Complex samples binary logistic regression was used to examine the associations between chronic disease and condition status and four movement behaviour outcomes: no regular transport-related walking, leisure time physical inactivity, muscle-strengthening activity on fewer than two days per week, and sitting or reclining for at least six hours per day. Each dependent variable was coded so that the less favourable movement behaviour represented the modelled outcome. Unadjusted associations were expressed as odds ratios (ORs), and adjusted associations as adjusted odds ratios (aORs), with 95% CIs.

For the categorical regression analyses, participants were classified as reporting no disease or condition, one disease or condition, or two or more diseases and conditions. An unadjusted model (Model 0) and four sequential multivariable models were fitted. Model 1 was adjusted for sex, age, educational attainment and BMI. Model 2 additionally included region of residence and household income quintile. Model 3 was further adjusted for functional mobility status, and Model 4 additionally included self-rated health.

In additional analyses based on Model 3, the number of reported diseases and conditions was first entered as a continuous variable without a quadratic term to assess linear trends in its association with each movement behaviour outcome. The resulting aOR represented the change in the odds of the outcome associated with each additional condition. Departure from linearity was subsequently examined in separate models in which the mean-centred linear term and its squared term were entered simultaneously. A statistically significant quadratic term was considered to indicate that the association could not be adequately characterised by a constant linear change in the odds. These analyses were adjusted for sex, age, educational attainment, BMI, region of residence, household income quintile and functional mobility status. Model 3 was selected for these analyses because sociodemographic characteristics and functional mobility were accounted for, whereas self-rated health was not included because it may partly reflect the number of chronic diseases and conditions and could therefore result in overadjustment.

Reference outcome categories were regular transport-related walking, any leisure time physical activity lasting at least 10 min, muscle-strengthening activity on at least two days per week, and sitting or reclining for less than six hours per day. For categorical exposure analyses, the reference category was no reported disease or condition. Reference categories for the covariates were male sex, age 15–29 years, higher education, BMI < 25.0 kg/m^2^, Belgrade region, the fifth (highest income) household income quintile, no difficulty with either functional mobility task, and very good/good self-rated health. An OR greater than 1 indicated higher odds of the modelled, less favourable movement behaviour relative to the relevant reference category.

Responses recorded as ‘do not know’, refusal, unavailable or otherwise missing were treated as missing and were not interpreted as indicating the absence of the characteristic under consideration. Chronic disease and condition status was determined only for participants with valid information on all 17 assessed conditions. Missing values were not imputed.

For each movement behaviour outcome, analyses were restricted to participants with complete information on the outcome and all variables included in Model 4. The same outcome-specific complete-case sample was retained across Models 0–4 so that differences between estimates were not attributable to changes in sample composition. The same samples were used for the linear trend and non-linearity analyses; although complete information on self-rated health was required for inclusion, self-rated health was not entered as a covariate in these models. Consequently, the unweighted analytical sample size differed between movement behaviour outcomes but remained constant across models within each outcome.

Overall associations and model effects were evaluated using design-adjusted Wald F tests. All statistical tests were two-sided, and *p*-values < 0.05 were considered statistically significant.

## 3. Results

### 3.1. Participants’ Characteristics

Among participants with complete information on all 17 assessed chronic diseases and conditions, 51.4% reported none, 18.0% reported one, 11.7% reported two, 7.6% reported three and 11.3% reported four or more. Women accounted for a slightly higher proportion of the study population than men (51.8% vs. 48.2%).

A pronounced age-related gradient was observed. The proportion reporting two or more chronic diseases and conditions increased from 3.4% among participants aged 15–29 years to 64.2% among those aged ≥65 years. Two or more conditions were also reported more frequently by women than by men (34.9% vs. 25.9%), whereas regional differences were comparatively modest.

Differences were also observed according to socioeconomic characteristics. The proportion reporting two or more chronic diseases and conditions was highest among participants with primary education or lower and decreased across successively higher levels of educational attainment. A less pronounced gradient was observed across household income quintiles, with the corresponding proportion generally decreasing from the lowest to the highest income quintile.

**Figure 1:**
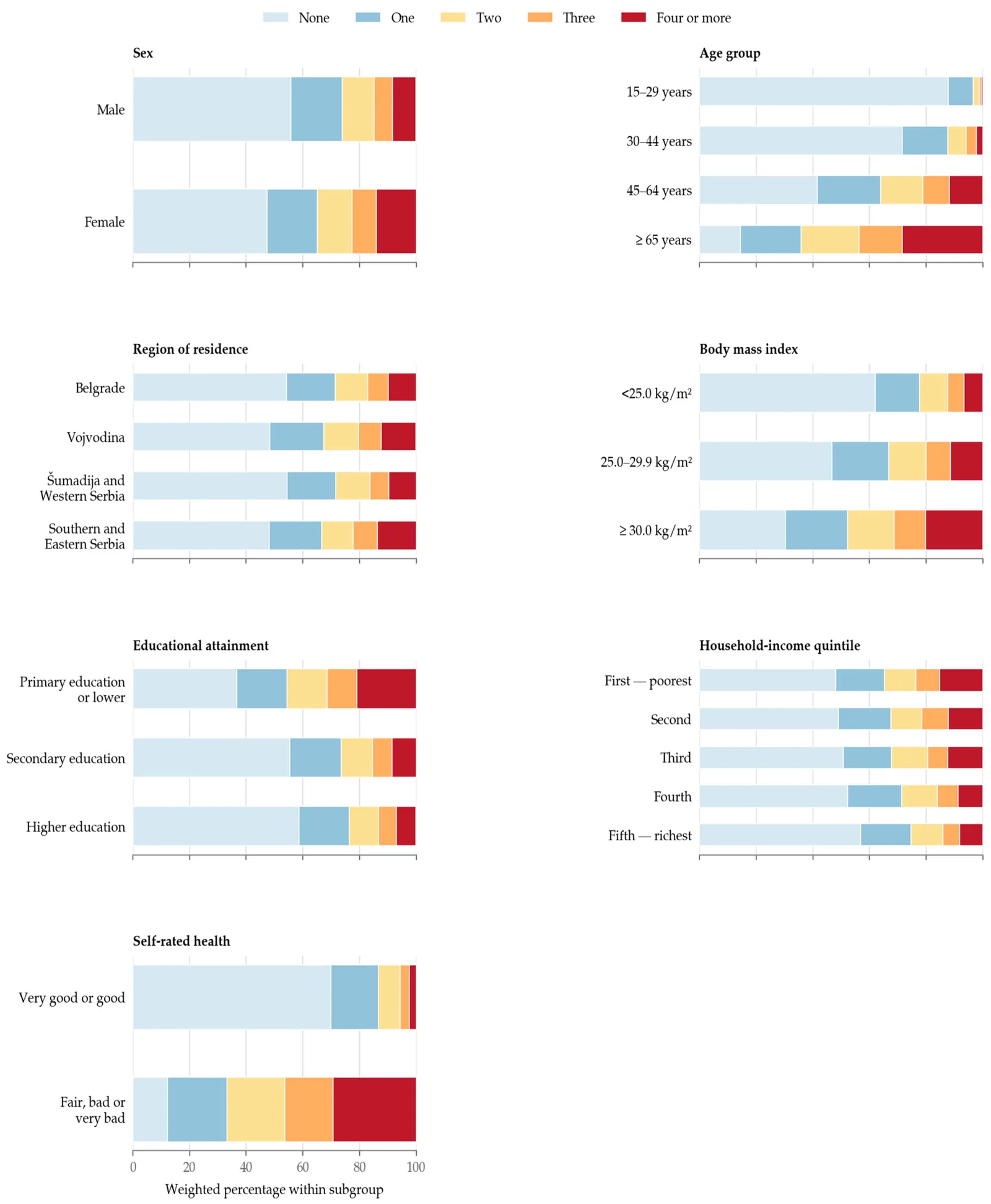
Distribution of the number of self-reported chronic diseases and conditions across participant characteristics. Percentages are survey-weighted and represent the distribution of chronic disease and condition categories within each subgroup. Differences in distributions were assessed using second-order Rao–Scott-adjusted Pearson chi-squared tests; all *p*-values were <0.001. Created in BioRender. Stanković, V. (2026) https://BioRender.com/xg1jp4x (accessed on 15 August 2026).

Chronic disease and condition status also differed across BMI categories and levels of self-rated health. The proportion reporting two or more conditions increased from 22.3% among participants with a BMI < 25.0 kg/m^2^ to 33.2% among those with a BMI of 25.0–29.9 kg/m^2^ and 47.8% among those with a BMI ≥ 30.0 kg/m^2^. A particularly marked difference was observed according to self-rated health: two or more conditions were reported by 13.3% of participants with very good or good self-rated health, compared with 66.8% of those reporting fair, bad or very bad health. The distribution of the number of chronic diseases and conditions differed significantly across all examined participant characteristics (all *p* < 0.001; Figure 1). Detailed survey-weighted estimates with 95% confidence intervals are provided in Table S1.

In addition to the differences observed across participant characteristics, movement behaviours and functional mobility also varied according to chronic disease and condition status. The prevalence of no regular transport-related walking, leisure time physical inactivity and muscle-strengthening activity on fewer than two days per week increased across categories of chronic disease and condition status (all *p* < 0.001). For sitting or reclining for at least six hours per day, the prevalence was lowest among participants with one condition and highest among those with two or more conditions (*p* = 0.001). Pronounced differences were also observed in functional mobility status: the proportion reporting no difficulty with either task decreased from 96.2% among participants with no condition to 52.9% among those with two or more conditions, whereas difficulty with both tasks increased from 2.8% to 36.5% (*p* < 0.001; Table 1). Complete results for all movement behaviour and functional mobility categories are presented in Table S2.

### 3.2. Associations Between Chronic Disease and Condition Status and Domain-Specific Movement Behaviours

In the unadjusted analyses, chronic disease and condition status was significantly associated with no regular transport-related walking, leisure time physical inactivity and muscle-strengthening activity on <2 days per week (all overall *p* < 0.001). Compared with participants reporting no chronic disease or condition, higher odds of each less favourable movement behaviour were observed among those reporting one condition and those reporting two or more conditions. For all three outcomes, the highest odds were observed among participants with two or more conditions.

Neither one nor two or more chronic diseases or conditions were individually associated with sitting or reclining for ≥6 h per day when compared with no reported condition. Nevertheless, the overall Wald F test indicated a statistically significant association between chronic disease and condition status and sedentary behaviour (Wald F = 3.37; *p* = 0.035).

The largest overall Wald F statistic was observed for leisure time physical inactivity (Wald F = 116.65), followed by muscle-strengthening activity on <2 days per week (Wald F = 67.53) and no regular transport-related walking (Wald F = 40.35). The overall association with sitting or reclining for ≥6 h per day was considerably weaker. Unweighted analytical sample sizes ranged from 10,218 to 10,537 across the four movement behaviour outcomes (Table 2).

After adjustment for sex, age, educational attainment and body mass index, significant overall associations were observed between chronic disease and condition status and no regular transport-related walking (Wald F = 7.21; *p* = 0.001), muscle-strengthening activity on <2 days per week (Wald F = 6.28; *p* = 0.002) and sitting or reclining for ≥6 h per day (Wald F = 5.70; *p* = 0.004). The overall association with leisure time physical inactivity did not reach statistical significance (Wald F = 2.97; *p* = 0.052).

Compared with participants reporting no chronic disease or condition, those reporting two or more conditions had higher odds of no regular transport-related walking (aOR 1.30, 95% CI 1.12–1.51), whereas no significant association was observed for one condition. Although the overall association with leisure time physical inactivity was not statistically significant, higher odds were observed for participants reporting two or more conditions (aOR 1.34, 95% CI 1.05–1.70). For muscle-strengthening activity on <2 days per week, higher odds were observed among participants reporting one condition (aOR 1.69, 95% CI 1.26–2.27), but not among those reporting two or more conditions. Participants reporting two or more conditions also had higher odds of sitting or reclining for ≥6 h per day (aOR 1.28, 95% CI 1.09–1.51), whereas the estimate for one condition was not statistically significant (Table 3).

Following additional adjustment for region of residence and household income quintile, significant overall associations were observed between chronic disease and condition status and all four movement behaviour outcomes: no regular transport-related walking (Wald F = 9.17; *p* < 0.001), leisure time physical inactivity (Wald F = 3.92; *p* = 0.020), muscle-strengthening activity on <2 days per week (Wald F = 7.60; *p* = 0.001) and sitting or reclining for ≥6 h per day (Wald F = 5.58; *p* = 0.004).

Compared with participants reporting no chronic disease or condition, those reporting two or more conditions had higher odds of no regular transport-related walking (aOR 1.35, 95% CI 1.17–1.56), leisure time physical inactivity (aOR 1.40, 95% CI 1.10–1.79) and sitting or reclining for ≥6 h per day (aOR 1.27, 95% CI 1.08–1.50). No significant associations with these outcomes were observed among participants reporting one condition. Conversely, higher odds of muscle-strengthening activity on <2 days per week were observed among participants reporting one condition (aOR 1.78, 95% CI 1.32–2.39), whereas the estimate for two or more conditions was not statistically significant (Table 4).

Following additional adjustment for functional mobility status, no significant overall associations were observed between chronic disease and condition status and no regular transport-related walking (Wald F = 0.41; *p* = 0.661), leisure time physical inactivity (Wald F = 1.95; *p* = 0.143), or sitting or reclining for ≥6 h per day (Wald F = 1.93; *p* = 0.146). Neither one condition nor two or more conditions were significantly associated with any of these outcomes when compared with no reported condition.

For muscle-strengthening activity on <2 days per week, the overall association remained statistically significant (Wald F = 7.00; *p* = 0.001). Compared with participants reporting no chronic disease or condition, higher odds were observed among those reporting one condition (aOR 1.75, 95% CI 1.30–2.35), whereas the estimate for two or more conditions was not statistically significant (aOR 1.15, 95% CI 0.85–1.57). Thus, following additional adjustment for functional mobility status, a significant association with chronic disease and condition status was retained only for muscle-strengthening activity on <2 days per week (Table 5).

Following additional adjustment for self-rated health, no significant overall associations were observed between chronic disease and condition status and no regular transport-related walking (Wald F = 0.11; *p* = 0.899), leisure time physical inactivity (Wald F = 0.20; *p* = 0.820), or sitting or reclining for ≥6 h per day (Wald F = 1.99; *p* = 0.138). Neither one condition nor two or more conditions were significantly associated with any of these outcomes when compared with no reported condition.

For muscle-strengthening activity on <2 days per week, the overall association remained statistically significant (Wald F = 5.13; *p* = 0.006). Compared with participants reporting no chronic disease or condition, higher odds were observed among those reporting one condition (aOR 1.60, 95% CI 1.18–2.16), whereas no significant association was observed among those reporting two or more conditions (aOR 0.93, 95% CI 0.67–1.30). Thus, after adjustment for all covariates included in Model 4, a significant association with chronic disease and condition status was retained only for muscle-strengthening activity on <2 days per week (Table 6).

When the number of chronic diseases and conditions was modelled as a continuous variable, a significant positive linear trend was identified only for sitting or reclining for ≥6 h per day. Each additional condition was associated with 4.5% higher odds of this outcome (aOR 1.045, 95% CI 1.006–1.087; *p* for linear trend = 0.025). No significant linear trends were identified for no regular transport-related walking (aOR 1.037, 95% CI 0.998–1.078; *p* = 0.063), leisure time physical inactivity (aOR 1.069, 95% CI 0.988–1.156; *p* = 0.095) or muscle-strengthening activity on <2 days per week (aOR 1.026, 95% CI 0.915–1.150; *p* = 0.661).

No evidence of departure from linearity was found for no regular transport-related walking (*p* for departure from linearity = 0.172), leisure time physical inactivity (*p* = 0.237) or sitting or reclining for ≥6 h per day (*p* = 0.755). By contrast, a significant quadratic term was identified for muscle-strengthening activity on <2 days per week (aOR 0.966, 95% CI 0.949–0.984; *p* < 0.001), indicating that its association with the number of chronic diseases and conditions could not be adequately characterised by a constant linear change in the odds (Table 7).

## 4. Discussion

In this nationally representative study of people aged ≥15 years in Serbia, the distribution of chronic diseases and conditions differed across all four movement behaviour domains. Less favourable movement behaviours were generally more common among participants reporting a greater number of conditions. However, the associations were attenuated after adjustment for sex, age, education, BMI, region of residence, household income and, particularly, functional mobility status, with self-rated health additionally included in the fully adjusted model. In that model, a significant overall association was retained only for muscle-strengthening activity on <2 days per week, with higher odds among participants reporting one condition but not among those reporting two or more conditions. After adjustment, there was little evidence of a consistent graded association between the number of chronic diseases and conditions and the movement behaviours examined.

The pronounced attenuation following adjustment for functional mobility indicates that part of the observed associations was statistically accounted for by differences in participants’ ability to walk 500 m and use stairs. The functional mobility measure captures reported difficulty with two everyday mobility tasks that are relevant to participation in several movement behaviours. However, the relationship between chronic conditions, functional mobility and movement behaviours is likely to be complex and potentially bidirectional. Longitudinal and systematic review evidence indicates that multimorbidity is associated with subsequent functional decline, while physical function has also been associated with the development and accumulation of chronic conditions [25,26]. As all variables were assessed concurrently, functional mobility cannot be interpreted as a mediator of the observed associations.

The unadjusted findings were broadly consistent with population-based studies reporting associations between chronic conditions or multimorbidity and lower physical activity [9,10,11]. A systematic review of objectively measured activity among people with multimorbidity reported generally low levels of moderate-to-vigorous physical activity and wide variation in adherence to physical activity recommendations, although the certainty of the evidence was very low [27]. More recently, a Danish accelerometry study found progressively lower adherence to the aerobic component of WHO physical activity recommendations as the number of conditions increased [28]. Conversely, a Swedish study of adults aged 50–64 years found only modest differences in objectively measured physical activity and sedentary time between people with and without chronic diseases after sociodemographic characteristics had been considered [29]. Complementing the available evidence, the present study examined four distinct movement behaviour domains and showed that their associations with the number of chronic diseases and conditions differed across domains and were markedly attenuated after functional mobility was taken into account.

An overall association between the number of chronic diseases and conditions and muscle-strengthening activity on <2 days per week was evident in the unadjusted model and remained statistically significant through Models 1–4 after sequential adjustment for the included covariates. However, the category-specific findings did not show a clear gradient: higher odds were observed among participants reporting one condition, whereas no significant association was found among those reporting two or more conditions. Consistent with these estimates, the significant quadratic term in the additional trend analysis based on Model 3 indicated a non-linear rather than consistently graded association.

Muscle-strengthening activity was uncommon in the study population, with only 5.6% of participants reporting such activity on at least two days per week. WHO recommendations identify muscle-strengthening activity as a distinct component of health-enhancing physical activity, while previous evidence has linked it to the preservation of physical function and lower risks of major non-communicable diseases and mortality [5,12].

For transport-related walking and leisure time physical inactivity, the significant unadjusted associations were no longer evident after functional mobility status was included in the models. In the present study, leisure time physical activity was defined as any participation lasting at least 10 min versus none and therefore did not distinguish between different frequencies, durations or intensities of activity.

The findings for sedentary behaviour varied according to how the number of chronic diseases and conditions was modelled. Although the overall association was significant in the unadjusted categorical model, none of the individual categories was significantly associated with sitting or reclining for ≥6 h per day in either the unadjusted or adjusted models. In the additional trend analysis based on Model 3, each additional condition was associated with 4.5% higher odds of sitting or reclining for ≥6 h per day, with no evidence of departure from linearity. This modest association was in the same direction as findings from middle-aged and older adults in low- and middle-income countries, in whom sedentary behaviour increased with the number of chronic conditions [30]. In the present study, sedentary behaviour was defined using a threshold of ≥6 h per day and did not distinguish between occupational, transport-related and leisure time sedentary behaviour. Sedentary behaviour is distinct from insufficient physical activity, as participation in physical activity does not necessarily imply limited sedentary time [5,15,16,17].

Overall, the associations between the number of chronic diseases and conditions and movement behaviours differed across the four domains and were substantially attenuated after adjustment, particularly when functional mobility was taken into account. The number of conditions alone therefore provided only a partial account of the differences in movement behaviours observed in this population. In the context of existing guidance, these findings highlight the relevance of both functional capacity and the specific movement behaviour domain examined. Current recommendations support regular physical activity and limiting prolonged sedentary behaviour among people living with chronic conditions where medically appropriate and functionally feasible [5]. From a public health perspective, the findings suggest that the number of chronic diseases and conditions alone may be insufficient for identifying people with less favourable movement behaviours. Differences across the examined domains, together with the attenuation observed after functional mobility was included, indicate that both functional mobility and the specific movement behaviour under consideration are relevant when population needs are assessed. These findings may help define more specific targets for further research and public health planning, but they do not establish the effectiveness of any particular intervention.

### Strengths and Limitations

Several strengths of this study should be noted. Data were obtained from a large, nationally representative survey of people aged ≥15 years and were collected using standardised EHIS methodology. All analyses accounted for the complex sampling design. For each movement behaviour outcome, the same complete-case analytical sample was used across Models 0–4, ensuring that changes in estimates across sequential models were not attributable to changes in sample composition.

Several limitations should be considered. First, the cross-sectional design precludes the determination of temporal order or causality. Chronic conditions may restrict movement behaviour, while less favourable movement behaviours may also contribute to the development or progression of chronic disease. Second, information on chronic diseases and conditions and movement behaviours was self-reported and may have been affected by recall error. The count assigned equal weight to all included chronic diseases and conditions, while information on their severity was unavailable. Third, complete-case analysis may have introduced selection bias, particularly because measured BMI was unavailable for a proportion of participants. Fourth, smoking and alcohol consumption were not included because of substantial missing information. Occupational circumstances were not included as a covariate, while type of settlement, although reflected in the survey stratification, was not available as a separate variable for inclusion in the regression models. Residual confounding related to these and other unmeasured or incompletely measured characteristics cannot be excluded. Fifth, although age was included in all adjusted models, age-stratified analyses were not performed, and possible differences in the examined associations across age groups were not evaluated. Finally, the threshold used to classify sitting or reclining for ≥6 h per day was selected for analytical purposes and should not be interpreted as an established clinical cut-off.

## 5. Conclusions

The variation in the associations across the examined domain-specific movement behaviours, together with their marked attenuation after functional mobility was taken into account, suggested that differences in movement behaviour could not be fully explained by the number of chronic diseases and conditions alone.

Functional mobility, self-rated health and sociodemographic characteristics were also relevant to the interpretation of movement behaviours, but longitudinal research remains necessary to clarify how these relationships develop over time and whether they differ according to the type, severity and duration of chronic conditions.

## Figures and Tables

**Table 1:** Survey-weighted prevalence of less favourable domain-specific movement behaviours and distribution of functional mobility status according to the number of self-reported chronic diseases and conditions.

Variables	Unweighted n		Number of Chronic Diseases and Conditions	
		None, % (95% CI)	One, % (95% CI)	Two or More, % (95% CI)
Domain-specific movement behaviour				
Transport-related walking	12,283			
No regular transport-related walking	5098	35.4 (32.5–38.3)	40.7 (37.6–43.9)	51.6 (49.0–54.1)
Leisure time physical activity	12,352			
No leisure time physical activity	10,841	79.2 (77.4–80.8)	90.1 (88.4–91.6)	95.0 (94.1–95.8)
Muscle-strengthening activity	12,347			
Muscle-strengthening activity on <2 days/week	11,653	90.1 (88.9–91.1)	96.5 (95.6–97.3)	97.4 (96.6–98.0)
Sedentary behaviour	11,790			
Sitting or reclining ≥ 6 h/day	3526	30.0 (27.5–32.7)	28.5 (25.7–31.5)	34.2 (31.7–36.7)
Functional mobility status	13,064			
No difficulty with either task	10,203	96.2 (95.4–96.8)	80.8 (78.7–82.8)	52.9 (50.9–55.0)
Difficulty with one task only	688	1.0 (0.7–1.4)	6.3 (5.3–7.6)	10.5 (9.4–11.8)
Difficulty with both tasks	2173	2.8 (2.3–3.5)	12.8 (11.3–14.5)	36.5 (34.7–38.4)

Values are unweighted counts and survey-weighted percentages with 95% confidence intervals (CIs). For the movement behaviour outcomes, percentages represent the prevalence of the respective less favourable movement behaviour within each chronic disease and condition category. For functional mobility status, percentages represent the distribution of functional mobility categories within each chronic disease and condition category and sum to approximately 100% within each column owing to rounding. Differences between chronic disease and condition categories were assessed using second-order Rao–Scott-adjusted Pearson chi-squared tests (*p* < 0.001 for transport-related walking, leisure time physical activity, muscle-strengthening activity and functional mobility status; *p* = 0.001 for sedentary behaviour). Analyses were based on available observations for each variable; consequently, unweighted sample sizes differed between domains.

**Table 2:** Unadjusted associations between the number of chronic diseases and conditions and domain-specific movement behaviours.

Variables	Domain-Specific Movement Behaviours							
	No Regular Transport-Related Walking OR (95% CI)	*p*	Leisure Time Physical Inactivity OR (95% CI)	*p*	Muscle-Strengthening Activity on <2 Days/Week OR (95% CI)	*p*	Sitting or Reclining ≥ 6 h/Day OR (95% CI)	*p*
Number of chronic diseases and conditions								
None	Reference	—	Reference	—	Reference	—	Reference	—
One	1.25 (1.09–1.43)	0.001	2.39 (1.95–2.92)	<0.001	3.22 (2.42–4.28)	<0.001	0.91 (0.78–1.05)	0.203
Two or more	1.79 (1.58–2.04)	<0.001	4.66 (3.78–5.73)	<0.001	3.76 (2.81–5.05)	<0.001	1.10 (0.96–1.26)	0.160
Overall effect of the number of chronic diseases and conditions, Wald F (df1, df2)	40.35 (2, 578)	<0.001	116.65 (2, 577)	<0.001	67.53 (2, 578)	<0.001	3.37 (2, 571)	0.035
Unweighted analytical n	10,481		10,537		10,533		10,218	

Model 0 was unadjusted. The reference exposure category was no self-reported chronic disease or condition among the 17 assessed. The reference outcome categories were regular transport-related walking, any leisure time physical activity lasting ≥10 consecutive minutes, muscle-strengthening activity on ≥2 days per week, and sitting or reclining for <6 h per day. ORs, 95% confidence intervals, Wald F statistics and *p*-values were estimated while accounting for the complex sampling design. For each movement behaviour outcome, the same outcome-specific complete-case analytical sample was used across Models 0–4. OR, odds ratio; CI, confidence interval.

**Table 3:** Adjusted associations between the number of chronic diseases and conditions and domain-specific movement behaviours: Model 1.

Variables	Domain-Specific Movement Behaviours							
	No Regular Transport-Related Walking aOR (95% CI)	*p*	Leisure Time Physical Inactivity aOR (95% CI)	*p*	Muscle-Strengthening Activity on <2 Days/Week aOR (95% CI)	*p*	Sitting or Reclining ≥ 6 h/Day aOR (95% CI)	*p*
Number of chronic diseases and conditions								
None	Reference	—	Reference	—	Reference	—	Reference	—
One	1.03 (0.90–1.19)	0.644	1.16 (0.93–1.46)	0.194	1.69 (1.26–2.27)	0.001	1.03 (0.88–1.20)	0.736
Two or more	1.30 (1.12–1.51)	0.001	1.34 (1.05–1.70)	0.017	1.20 (0.88–1.62)	0.244	1.28 (1.09–1.51)	0.003
Overall effect of the number of chronic diseases and conditions, Wald F (df1, df2)	7.21 (2, 578)	0.001	2.97 (2, 577)	0.052	6.28 (2, 578)	0.002	5.70 (2, 571)	0.004
Unweighted analytical n	10,481		10,537		10,533		10,218	

Model 1 was adjusted for sex, age, educational attainment and body mass index. The reference exposure category was no self-reported chronic disease or condition among the 17 assessed. The reference outcome categories were regular transport-related walking, any leisure time physical activity lasting ≥10 consecutive minutes, muscle-strengthening activity on ≥2 days per week, and sitting or reclining for <6 h per day. aORs, 95% confidence intervals, Wald F statistics and *p*-values were estimated with adjustment for the complex sampling design. For each movement behaviour outcome, the same outcome-specific complete-case analytical sample was used across Models 0–4. aORs, 95% confidence intervals, Wald F statistics and *p*-values were estimated while accounting for the complex sampling design.

**Table 4:** Adjusted associations between the number of chronic diseases and conditions and domain-specific movement behaviours: Model 2.

Variables	Domain-Specific Movement Behaviours							
	No Regular Transport-Related Walking aOR (95% CI)	*p*	Leisure Time Physical Inactivity aOR (95% CI)	*p*	Muscle-Strengthening Activity on <2 Days/Week aOR (95% CI)	*p*	Sitting or Reclining ≥ 6 h/Day aOR (95% CI)	*p*
Number of chronic diseases and conditions								
None	Reference	—	Reference	—	Reference	—	Reference	—
One	1.05 (0.92–1.21)	0.472	1.22 (0.97–1.53)	0.097	1.78 (1.32–2.39)	<0.001	1.02 (0.87–1.20)	0.812
Two or more	1.35 (1.17–1.56)	<0.001	1.40 (1.10–1.79)	0.007	1.26 (0.93–1.70)	0.140	1.27 (1.08–1.50)	0.004
Overall effect of the number of chronic diseases and conditions, Wald F (df1, df2)	9.17 (2, 578)	<0.001	3.92 (2, 577)	0.020	7.60 (2, 578)	0.001	5.58 (2, 571)	0.004
Unweighted analytical n	10,481		10,537		10,533		10,218	

Model 2 was adjusted for sex, age, educational attainment, body mass index, region of residence and household income quintile. The reference exposure category was no self-reported chronic disease or condition among the 17 assessed. The reference outcome categories were regular transport-related walking, any leisure time physical activity lasting ≥10 consecutive minutes, muscle-strengthening activity on ≥2 days per week, and sitting or reclining for <6 h per day. aORs, 95% confidence intervals, Wald F statistics and *p*-values were estimated with adjustment for the complex sampling design. For each movement behaviour outcome, the same outcome-specific complete-case analytical sample was used across Models 0–4. aORs, 95% confidence intervals, Wald F statistics and *p*-values were estimated while accounting for the complex sampling design.

**Table 5:** Adjusted associations between the number of chronic diseases and conditions and domain-specific movement behaviours: Model 3.

Variables	Domain-Specific Movement Behaviours							
	No Regular Transport Walking aOR (95% CI)	*p*	Leisure Time Physical Inactivity aOR (95% CI)	*p*	Muscle-Strengthening Activity on <2 Days/Week aOR (95% CI)	*p*	Sitting or Reclining ≥ 6 h/Day aOR (95% CI)	*p*
Number of chronic diseases and conditions								
None	Reference	—	Reference	—	Reference	—	Reference	—
One	1.00 (0.87–1.16)	0.980	1.19 (0.94–1.50)	0.152	1.75 (1.30–2.35)	<0.001	1.01 (0.85–1.19)	0.947
Two or more	1.07 (0.91–1.25)	0.429	1.25 (0.98–1.61)	0.078	1.15 (0.85–1.57)	0.371	1.16 (0.98–1.37)	0.093
Overall effect of the number of chronic diseases and conditions, Wald F (df1, df2)	0.41 (2, 578)	0.661	1.95 (2, 577)	0.143	7.00 (2, 578)	0.001	1.93 (2, 571)	0.146
Unweighted analytical n	10,481		10,537		10,533		10,218	

Model 3 was adjusted for sex, age, educational attainment, body mass index, region of residence, household income quintile and functional mobility status. The reference exposure category was no self-reported chronic disease or condition among the 17 assessed. The reference outcome categories were regular transport-related walking, any leisure time physical activity lasting ≥10 consecutive minutes, muscle-strengthening activity on ≥2 days per week, and sitting or reclining for <6 h per day. aORs, 95% confidence intervals, Wald F statistics and *p*-values were estimated while accounting for the complex sampling design. For each movement behaviour outcome, the same outcome-specific complete-case analytical sample was used across Models 0–4. aOR, adjusted odds ratio; CI, confidence interval.

**Table 6:** Adjusted associations between the number of chronic diseases and conditions and domain-specific movement behaviours: Model 4.

Variables	Domain-Specific Movement Behaviours							
	No Regular Transport-Related Walking aOR (95% CI)	*p*	Leisure Time Physical Inactivity aOR (95% CI)	*p*	Muscle-Strengthening Activity on <2 Days/Week aOR (95% CI)	*p*	Sitting or Reclining ≥ 6 h/Day aOR (95% CI)	*p*
Number of chronic diseases and conditions								
None	Reference	—	Reference	—	Reference	—	Reference	—
One	0.97 (0.84–1.12)	0.687	1.08 (0.85–1.37)	0.543	1.60 (1.18–2.16)	0.002	1.01 (0.86–1.20)	0.878
Two or more	1.00 (0.84–1.18)	0.979	1.01 (0.78–1.31)	0.942	0.93 (0.67–1.30)	0.670	1.18 (0.98–1.41)	0.085
Overall effect of the number of chronic diseases and conditions, Wald F (df1, df2)	0.11 (2, 578)	0.899	0.20 (2, 577)	0.820	5.13 (2, 578)	0.006	1.99 (2, 571)	0.138
Unweighted analytical n	10,481		10,537		10,533		10,218	

Model 4 was adjusted for sex, age, educational attainment, body mass index, region of residence, household income quintile, functional mobility status and self-rated health. The reference exposure category was no self-reported chronic disease or condition among the 17 assessed. The reference outcome categories were regular transport-related walking, any leisure time physical activity lasting ≥10 consecutive minutes, muscle-strengthening activity on ≥2 days per week, and sitting or reclining for <6 h per day. aORs, 95% confidence intervals, Wald F statistics and *p*-values were estimated while accounting for the complex sampling design. For each movement behaviour outcome, the same outcome-specific complete-case analytical sample was used across Models 0–4. aOR, adjusted odds ratio; CI, confidence interval.

**Table 7:** Linear trends and tests for departure from linearity in the associations between the number of chronic diseases and conditions and domain-specific movement behaviours.

Domain-Specific Movement Behaviour	Linear Trend, aOR per Additional per Additional Chronic Disease or Condition (95% CI)	*p* for Linear Trend	Quadratic Term for the Number of Chronic Diseases and Conditions, aOR (95% CI)	*p* for Departure from Linearity
No regular transport-related walking	1.037 (0.998–1.078)	0.063	1.008 (0.997–1.019)	0.172
Leisure time physical inactivity	1.069 (0.988–1.156)	0.095	0.988 (0.967–1.008)	0.237
Muscle-strengthening activity on <2 days per week	1.026 (0.915–1.150)	0.661	0.966 (0.949–0.984)	<0.001
Sitting or reclining ≥ 6 h/day	1.045 (1.006–1.087)	0.025	1.002 (0.991–1.012)	0.755

Linear trend estimates were obtained from separate models in which the number of chronic diseases and conditions was entered as a continuous variable; the aOR represents the change in the odds of the outcome associated with each additional condition. Departure from linearity was assessed in separate models in which the mean-centred linear and quadratic terms were entered simultaneously. The reported *p*-value for the quadratic term represents the test for departure from linearity. All models were adjusted for sex, age, educational attainment, body mass index, region of residence, household income quintile and functional mobility status (Model 3). The same outcome-specific complete-case analytical samples as those used across Models 0–4 were retained: no regular transport-related walking, *n* = 10,481; leisure time physical inactivity, *n* = 10,537; muscle-strengthening activity on <2 days per week, *n* = 10,533; and sitting or reclining for ≥6 h per day, *n* = 10,218. The reference outcome categories were regular transport-related walking, any leisure time physical activity lasting ≥10 consecutive minutes, muscle-strengthening activity on ≥2 days per week, and sitting or reclining for <6 h per day. Estimates and *p*-values were calculated while accounting for the complex sampling design. aOR, adjusted odds ratio; CI, confidence interval.

## Data Availability

Due to privacy or ethical restrictions, the data are not available. The database was officially transferred by the Institute of Public Health of Serbia “ Dr. Milan Jovanović Batut”, the current holder of the data rights, to the University of Kragujevac by official letter for further research.

## Supplementary Materials

The following supporting information can be downloaded at: https://www.mdpi.com/article/10.3390/healthcare14172783/s1, Table S1: Distribution of the number of self-reported chronic diseases and conditions across characteristics of the study participants; Table S2: Survey-weighted distribution of domain-specific movement behaviours and functional mobility status according to the number of self-reported chronic diseases and conditions.
