# Supplementary material for: The Number of Chronic Conditions and Domain-Specific Movement Behaviours Among People Aged ≥15 Years: Findings from the 2019 Serbian National Health Survey
Source: Healthcare (Basel). 2026 Sep 1;14(17):2783. doi: 10.3390/healthcare14172783 (PMC13565304; doi:10.3390/healthcare14172783)
Supplement: Supplementary file 1 [file healthcare-14-02783-s001.zip › healthcare-4447812-supplementary.pdf]

MDPI

healthcare

Supplementary Materials

The Number of Chronic Conditions and Domain-Specific Movement Behaviours among People Aged ≥15 Years: Findings from the 2019 Serbian National Health Survey

Table S1. Distribution of the number of self-reported chronic diseases and conditions across characteristics of the study participants.

Variables	Unweighted n	Total, % (95% CI)	Number of chronic diseases and conditions				
			None, % (95% CI)	One, % (95% CI)	Two, % (95% CI)	Three, % (95% CI)	Four or more, % (95% CI)
Sex	13,076						
Male	6382	48.2 (47.5–48.9)	55.8 (54.2–57.3)	18.2 (17.2–19.4)	11.1 (10.3–12.0)	6.5 (5.9–7.2)	8.3 (7.6–9.1)
Female	6694	51.8 (51.1–52.5)	47.4 (45.8–49.0)	17.7 (16.6–18.9)	12.3 (11.4–13.2)	8.6 (7.9–9.5)	14.0 (12.9–15.2)
Age group	13,076						
15–29 years	2307	19.4 (18.5–20.4)	87.9 (86.1–89.4)	8.7 (7.4–10.3)	2.1 (1.5–3.0)	0.8 (0.5–1.5)	0.5 (0.2–0.9)
30–44 years	2780	24.5 (23.5–25.6)	71.5 (69.0–73.9)	16.2 (14.6–17.9)	6.5 (5.5–7.8)	3.5 (2.7–4.5)	2.3 (1.7–3.1)
45–64 years	4335	32.1 (31.2–33.0)	41.6 (39.6–43.7)	22.4 (21.0–23.9)	14.9 (13.7–16.2)	9.3 (8.3–10.4)	11.8 (10.5–13.1)
≥65 years	3654	24.0 (23.0–25.0)	14.4 (13.0–15.9)	21.4 (19.7–23.1)	20.5 (19.0–22.1)	15.2 (13.9–16.5)	28.5 (26.5–30.7)
Region of residence	13,076						
Belgrade	3047	24.2 (22.9–25.6)	54.3 (51.5–57.0)	17.2 (15.6–19.0)	11.4 (10.0–12.9)	7.2 (6.1–8.5)	9.9 (8.4–11.6)
Vojvodina	2928	26.6 (25.1–28.1)	48.3 (45.9–50.7)	19.2 (17.5–21.0)	12.1 (10.7–13.7)	8.1 (7.2–9.1)	12.2 (10.7–14.0)
Šumadija and Western Serbia	4211	27.6 (26.4–28.7)	54.5 (52.1–56.9)	17.1 (15.8–18.5)	12.0 (10.8–13.2)	6.8 (5.9–7.8)	9.6 (8.4–11.0)
Southern and Eastern Serbia	2890	21.6 (20.4–22.9)	48.1 (45.1–51.0)	18.5 (16.6–20.5)	11.2 (10.0–12.5)	8.6 (7.3–10.0)	13.7 (11.8–15.8)

Body mass index category	10,615						
<25.0 kg/m²	4418	43.0 (41.7–44.3)	62.0 (60.2–63.8)	15.7 (14.4–17.0)	10.0 (9.0–11.1)	5.7 (5.0–6.6)	6.6 (5.8–7.5)
25.0–29.9 kg/m²	3898	36.3 (35.2–37.5)	46.7 (44.6–48.7)	20.2 (18.7–21.8)	13.0 (11.9–14.1)	8.6 (7.6–9.7)	11.6 (10.4–12.9)
≥30.0 kg/m²	2299	20.7 (19.8–21.7)	30.4 (28.1–32.8)	21.8 (19.9–23.7)	16.6 (14.9–18.5)	10.9 (9.6–12.3)	20.3 (18.3–22.5)
Educational attainment	13,070						
Primary education or lower	3462	24.5 (23.1–26.0)	36.7 (34.4–39.0)	17.7 (16.2–19.3)	14.2 (12.8–15.8)	10.5 (9.4–11.7)	20.9 (19.1–22.8)
Secondary education	7254	55.4 (53.9–56.9)	55.3 (53.7–56.9)	18.3 (17.2–19.4)	11.1 (10.2–11.9)	6.8 (6.2–7.5)	8.6 (7.8–9.4)
Higher education	2354	20.1 (18.8–21.4)	58.7 (56.1–61.2)	17.6 (15.9–19.5)	10.4 (9.1–12.0)	6.3 (5.3–7.6)	6.9 (5.9–8.2)
Household income quintile	13,076						
First—poorest	2677	19.4 (17.8–21.2)	48.0 (45.6–50.4)	17.3 (15.7–19.0)	11.1 (9.8–12.6)	8.3 (7.2–9.5)	15.3 (13.6–17.2)
Second	2669	20.2 (18.7–21.7)	49.1 (46.5–51.7)	18.5 (16.9–20.3)	10.9 (9.7–12.2)	9.4 (8.2–10.8)	12.1 (10.7–13.7)
Third	2649	19.8 (18.4–21.2)	50.7 (48.1–53.3)	17.1 (15.5–18.8)	12.8 (11.3–14.4)	7.1 (6.0–8.3)	12.3 (10.8–14.0)
Fourth	2628	20.1 (18.6–21.6)	52.3 (49.9–54.6)	19.1 (17.3–21.0)	12.5 (11.1–14.1)	7.4 (6.3–8.6)	8.7 (7.5–10.2)
Fifth—richest	2453	20.5 (19.0–22.1)	56.8 (54.1–59.4)	17.9 (16.3–19.7)	11.2 (9.9–12.7)	6.0 (5.1–7.2)	8.1 (6.8–9.5)
Self-rated health	12,373						
Very good or good	7944	66.8 (65.5–68.0)	69.9 (68.5–71.4)	16.8 (15.8–17.8)	7.7 (7.0–8.5)	3.2 (2.8–3.7)	2.4 (2.0–2.8)
Fair, bad, or very bad	4429	33.2 (32.0–34.5)	12.3 (11.0–13.7)	20.9 (19.3–22.5)	20.5 (19.1–22.1)	17.0 (15.7–18.4)	29.3 (27.4–31.4)

Values are survey-weighted percentages with 95% confidence intervals (CIs); all sample sizes (*n*) are unweighted. Total percentages represent the weighted proportion of participants in each category of the respective characteristic, whereas percentages under 'Number of chronic diseases and conditions' represent the weighted distribution of chronic disease and condition categories within each subgroup. Unweighted sample sizes differ across characteristics because of missing data. Differences in distributions were assessed using second-order Rao–Scott-adjusted Pearson chi-squared tests; all p-values were <0.001.

Table S2. Survey-weighted distribution of domain-specific movement behaviours and functional mobility status according to the number of self-reported chronic diseases and conditions.

Variables	Un-weighted n	None, % (95% CI)	One, % (95% CI)	Two or more, % (95% CI)	p
**Transport-related walking**	12,283				<0.001
Regular transport-related walking: ≥5 days/week and ≥30 min/day	7185	64.6 (61.7–67.5)	59.3 (56.1–62.4)	48.4 (45.9–51.0)	
No regular transport-related walking	5098	35.4 (32.5–38.3)	40.7 (37.6–43.9)	51.6 (49.0–54.1)	
Leisure time physical activity	12,352				<0.001
Any leisure time physical activity in bouts ≥10 min	1511	20.8 (19.2–22.6)	9.9 (8.4–11.6)	5.0 (4.2–5.9)	
No leisure time physical activity	10,841	79.2 (77.4–80.8)	90.1 (88.4–91.6)	95.0 (94.1–95.8)	
Muscle-strengthening activity	12,347				<0.001
Muscle-strengthening activity on ≥2 days/week	694	9.9 (8.9–11.1)	3.5 (2.7–4.4)	2.6 (2.0–3.4)	
Muscle-strengthening activity on <2 days/week	11,653	90.1 (88.9–91.1)	96.5 (95.6–97.3)	97.4 (96.6–98.0)	
Sedentary behaviour	11,790				0.001
Sitting or reclining <6 h/day	8264	70.0 (67.3–72.5)	71.5 (68.5–74.3)	65.8 (63.3–68.3)	
Sitting or reclining ≥6 h/day	3526	30.0 (27.5–32.7)	28.5 (25.7–31.5)	34.2 (31.7–36.7)	
**Functional mobility status**	13,064				<0.001
No difficulty with either task	10,203	96.2 (95.4–96.8)	80.8 (78.7–82.8)	52.9 (50.9–55.0)	
Difficulty with one task only	688	1.0 (0.7–1.4)	6.3 (5.3–7.6)	10.5 (9.4–11.8)	
Difficulty with both tasks	2173	2.8 (2.3–3.5)	12.8 (11.3–14.5)	36.5 (34.7–38.4)	

Values are unweighted counts and survey-weighted percentages with 95% confidence intervals (CIs). Percentages represent the distribution of movement behaviour or functional mobility categories within each chronic disease and condition category and sum to approximately 100% within each domain and column owing to rounding. Differences between chronic disease and condition categories were assessed using second-order Rao–Scott-adjusted Pearson chi-squared tests. Analyses were based on available observations for each variable; consequently, unweighted sample sizes differed between domains.
